# Green Tea Seed Isolated Saponins Exerts Antibacterial Effects against Various Strains of Gram Positive and Gram Negative Bacteria, a Comprehensive Study* In Vitro* and* In Vivo*

**DOI:** 10.1155/2018/3486106

**Published:** 2018-11-26

**Authors:** Muhammad Imran Khan, Abdulatef Ahhmed, Jin Hyuk Shin, Jun Soo Baek, Min Yong Kim, Jong Deog Kim

**Affiliations:** ^1^Department of Biotechnology, Chonnam National University, San 96-1, Dun-Duk Dong, Yeosu, Chonnam, 550-749, Republic of Korea; ^2^Department of Food Engineering, Davutpasa Campus, Esenler, Istanbul 34210, Turkey; ^3^Department of Refrigeration Engineering, Chonnam National University, San 96-1, Dun- Duk Dong, Yeosu, Chonnam 550-749, Republic of Korea; ^4^Research Center on Anti-Obesity and Health Care, Chonnam National University, San 96-1, Dun-Duk Dong, Yeosu, Chonnam, 550-749, Republic of Korea

## Abstract

Bacteria are one of the major causes of severe infections and diseases of plants and animals.* Salmonella *are crucially important due to infection in poultry leading to huge economical loses. Due to high cost and microbial resistance to the currently available chemical antibiotics, demand of screening natural products with antibiotics effects is increased. Plants are rich sources of natural bioactive compounds with antibiotic effects. Saponins are natural compounds of plant sources having a diverse range of applications. In present study we investigated the* in vitro* and* in vivo* antibacterial activities of green tea seed extracted saponins. Green tea seeds crude extract was prepared in 70% ethanol by continuous reflux in heating mantel for 5 hours. Crude saponins were extracted from the crude ethanolic extract of green tea seed by column chromatography using macroporous resin (D101). Saponin mixture in fraction 1 (Fr1) was obtained from crude saponins extract via column chromatography. Fr2 and Fr3 were isolated from saponins mixture by preparative HPLC. Antibacterial activities of the isolated saponins fractions were investigated against* Escherichia coli* (ATCC 25922),* Streptococcus aureus *(ATCC 12600), and six serovars of* Salmonella. In vitro* antibacterial activities were determined by disc-diffusion method and growth inhibition in liquid culture using 96-well plate. Results showed that the green tea isolated saponins fractions possess antibacterial effects in the following order Fr1>Fr2>Fr3. Antibacterial mechanism of saponins was elucidated by cell wall and membrane damaging potential of saponins determined by measuring AKP and soluble proteins levels. Fr1 was further used for* in vivo* antibacterial activities. Five-week grown chickens were selected for* in vivo* work, divided into three groups as control, infected, and treatment groups. Infected and treatment groups chickens were infected with bacteria and only treatment group chickens were treated with saponins. The qRT- PCR analysis of the blood and feces samples of the different groups' animals shows the presence of bacteria only in infected group while reduced expression levels of the bacterial pathogens were found in the samples of treatment group. Our results demonstrated that the green tea seed saponins used in this study possess strong antibacterial activities.

## 1. Introduction

Bacteria are one of the major causes of various severe infectious diseases in plants and animal including human being. There are a huge number of pathogenic bacteria belonging to different genera causing infection and disease in different hosts. The rod-shaped Gram-negative bacteria* Salmonella* is responsible for salmonellosis and zoonosis. Various Salmonella species cause infection in poultry and resulted in huge economic losses [1].* Salmonella* serotypes are the cause of* Salmonella* infections, a worldwide major public health concern. Avian infection in poultry occurred by different species of Genus* Salmonella* [2].* Salmonella* make frequent infection by salmonellosis and zoonosis in poultry populations [3, 4]. The presence of* Salmonella* in poultry animals is a major risk, making the transmission of bacteria to eggs and meat which can be forward to human infection [5]. Antibacterial agents are necessary for the treatment of bacterial disease and infection; however the synthetic nature, adverse side effects, and the emerged issue of microbial resistance due to increased use have led to the need of screening novel antibacterial products with new targets and natural origins [6]. Majority of the antibiotics from microbial natural products have limited efficacy as not well annotated [7, 8].

Plants have an amazing ability to produce a wide variety of secondary metabolites, like alkaloids, glycosides, terpenoids, saponins, steroids, flavonoids, tannins, quinones, and coumarins [9]. These biomolecules are the source of plant-derived antimicrobial substances (PDAms) [10]. Some natural products are highly efficient in the treatment of bacterial infections [11].* Camellia seeds* are rich source of bioactive compounds including polysaccharides, proteins, saponins, flavonoids, and unsaturated fatty acids and have been reported to possess bioactivities such as anti-inflammatory, anticancer, antioxidant, antiobesity, antiangiogenic, foamable, hepatoprotective, insecticidal, etc. [12–25]

Saponins are natural compounds of a diverse group generally consists of sugar (glycon) and nonsugar part (aglycon) connected by a glyosidic linkage. The aglycone part is also called sapoginins which may be either steroidal (C27) or a triterpenoid (C30) [26] and attached to one or more glycone moieties which may be hexoses and pentoses. The biological properties such as antibacterial, anti-inflammatory, antifungal, and antiviral activities of various saponins are based on their chemical structures [27–29]. Saponins bind with cholesterol inside cell making the saponin-cholesterol complex which results finally in lysing of the cells [30]. Saponins disturbed the permeability of bacterial cells by binding to the outer membrane [31, 32].

The elimination of pathogenic Gram-positive and Gram-negative bacterial strains is an important medical problem and can be achieved by bacteria lysis, inhibition of bacterial adhesion, and increasing the permeability of bacterial cell walls [33–35].

Present study was conducted to investigate the antibacterial effects of green tea seed extracted saponins* in vitro* and* in vivo* with confirmation of the lysis potential and detection of the pathogens by qRT-PCR analysis.

## 2. Materials and Methods

### 2.1. Extraction and Purification

Green tea (*Camellia sinensis*) seeds were collected from the Myungin Shin Gwang Su tea garden (Suncheon, Korea). The seeds were dried, dehulled, and ground into powder. The powder (3 kg) was defatted with n-hexane (4 L) under sonication at 30°C for 5 h and then dried. The defatted seed powder was further extracted by refluxing with 70% ethanol at 60°C for 5 hr. The resulting extract was filtered, concentrated using a rotatory vacuum evaporator (SB-100, Eyela), freeze dried, and weighed. The extract was again subjected to extraction with butanol and water mixture and concentrated with rotary rotatory evaporator. Saponins extraction from the crude extract was carried out by nonpolar macropours resins (D101). Resins were thoroughly washed 2 times with ethanol and then distilled water. 10 g of the extract was dissolved in 30 ml double distilled water and mixed with the washed resin and kept overnight at room temperature. The mixture of the extract and resin was the loaded to a column of 500 ml and eluted with 0.3 N NaOH resulted in a dark radish brown fraction. The resins and the fraction were then neutralized, mixed, and incubated overnight in the column again and then eluted with 100% ethanol resulting again in brownish color containing crude saponins. The crude saponins were then subjected to column chromatography using C-18 Luna column. The obtained saponins fraction was termed as Fr1 contained different types of saponins. Fr1 was further fractioned into Fr2 and Fr3 by HPLC. Analysis of the saponins fractions was done by HPLC-MS for detection of the presence of various saponins.

### 2.2. Thin-Layer Chromatography

Thin-layer chromatography (TLC) was performed to analyze the saponins fractions. 10 mg/mL of the all the samples was dissolved in 80% ethanol. 10 mL of each sample was applied to a normal-phase TLC plate (TLC Silica gel 60, glass plates 10 × 20 cm, Merck, Germany), and n-butanol, water, and acetic (84:14:17) were used as development solution. The developed plates were visualized by dipping in 10% H_2_SO_4_ and heated in an oven at 115°C for 13 min to visualize the saponin bands.

### 2.3. In Vitro Antibacterial Activities

The* in vitro* antibacterial activities of saponin fractions were investigated against various* Salmonella *serotypes,* Escherichia coli* (Gram-negative), and* Staphylococcus aureus* (Gram-positive). All the strains were bought from American Type Culture Collection (ATCC). The strains used were* Escherichia coli* (ATCC 25922),* Salmonella typhimurium* (ATCC 14028),* Salmonella enteritidis* (ATCC 13076),* Salmonella gallinarum* (ATCC 9184),* Salmonella choleraesuis* (ATCC 7001),* Salmonella pullorum* (ATCC 19945),* Salmonella dublin* (ATCC 15480), and* Staphylococcus aureus *(ATCC 12600). Disc-diffusion method was used as a preliminary test and then the antibacterial activities were also determined by bacteria viability and lethality in the pressence of verious concentrations of saponin fractions [21]. The inoculum of bacteria was prepared in Luria–Bertani broth (Difco, USA) and nurturant broth (Difco) (2 different growth media were tested individually for best growth and inoculum preparation) and then 500 *μ*L active culture of each strain was spread on the respective Muller-Hinton agar (BD biosciences) plates. Then, filter paper discs (Advantech, Japan) impregnated with saponin fractions (1mg/ml) were placed on the surfaces of the plates. Ampicillin (MP Biomedical, France) was used as positive control. All plates were incubated at 37 ± 1°C for 24 h and after that the inhibition zones (IZ) were measured of each strain.

The antibacterial activities of the saponin isolated fractions were also determined by using bacteria viability in 96-well plates. Bacteria were grown in their respective liquid culture and 20 *μ*L of active cultures of each organism was inoculated into 100 *μ*L nutrient broth (Difco) per well of the 96-well plate. Then various concentrations of the saponin fractions were added to the respective plate wells and total volume per well were adjusted up to 200 *μ*L. Plates were kept in an incubator for 37 ± 1°C for 24 h. After that the growth of each organism was measured by checking the optical density (OD) at a wavelength of 660 nm using a microplate reader. Bacteria without treatment with saponins were used as reference.

#### 2.3.1. Minimum Inhibitory Concentrations (MIC)

The minimum inhibitory concentrations (MIC) of saponins fractions were determined as previously described [21]. In brief bacteria were grown in their respective liquid culture and 20 *μ*L of active cultures of each organism was inoculated into 100 *μ*L nutrient broth (Difco) per well of the 96-well plate. Then various concentrations of the saponin mixture and fractions were added to the respective plate wells and total volume per well were adjusted up to 200 *μ*L. Plates were kept in an incubator for 37 ± 1°C for 24 h. After that 20 *μ*L of 0.2 mg/mL INT {2-(4-Iodophenyl)-3-(4-nitrophenyl)-5-phenyl-2H-tetrazolium chloride} was added to each well, then the plates were incubated for four more h at 37 ± 1°C. Absorbance was measured at a wavelength of 600 nm using a microplate reader.

### 2.4. Bacterial Cell Wall Damaging Potential of Saponins

For determination the lysis of bacterial cell by saponins we measured the AKP (Alkaline phosphatase) contents of each strain after exposing to saponins. AKP contents were measured according to the method of He et al., with slight modifications [36]. AKP is found between the cell wall and cell membrane of bacteria cell. The AKP leakage occurs only when cell wall of the bacteria is damage. Hence the amount of AKP is directly proportion to the cell lysis.

Fresh bacterial cultures were prepared for each strain. Saponins (Fr1) at the concentration of 100 ug/ml and MIC were mixed with 5 × 10^6^ CFU of each strain and incubated in a shaking incubator at 37°C strain. Bacterial suspension without treatment with saponins was used as negative control group. Samples were taken at zero time and after every 1 hour. Samples were centrifuged at 3000 rpm for 20 min. Supernatant was used for measuring ALP contents using AKP kit.

### 2.5. Determination of Soluble Protein Contents/Membrane Integrity Assay

To determine the bacteria lysing potential of saponins, concentration of soluble protein in bacterial suspension was measured after treatment with saponins at the concentration of MIC. Bacterial suspension without treatment was taken as negative control. After addition of saponins to the bacterial suspension (5 × 10^6^ CFU of each strain) the samples were incubated for 15 minutes and then absorbance of each group was check at 595 nm using spectrophotometer.

### 2.6. *In Vivo* Antibacterial Assay

For* in vivo *investigation of antibacterial potential of the green tea seed saponins fraction Fr1 (crude saponins) was used due to its relatively high* in vitro* antimicrobial activities. Chickens were used as model animals. Animals used were treated according to the guide for the care and use of National Institutes of Health Laboratory and the experimental protocol was approved by the Chonnam National University Ethical Community for Animal studies (Approval No. CNU IACUC–YS–2018-1). First safe doses of saponins were determined by MTT assay using different eukaryotic cell lines. The saponins concentration used for* in vivo* antibacterial experiments was less than that of the highest safe concentration determined by cell viabilities assays* in vitro* and* in vivo.* 5-week grown chickens were divided into three groups for each bacteria type (n=5), i.e., control group (chicken was not infected with the respective bacteria given saponins), infection group (chicken was infected with the respective bacteria but not treated with saponins), and infection and treatment group (chicken was infected with the respective bacteria and treated with saponins). Chickens of each group were kept in separate places in control conditions. All animals of the control group were kept in one area while that of infected and treatment group was separated for each bacteria strain. The area was completely fenced, and the animals of each group had no access to other groups. Also, all the chickens were labeled for identification.

Bacteria were administered orally (100*μ*L active culture) to the chicken of respective group. Saponins (Fr1) 1 mg/chicken in distilled water were orally injected to the chicken of treatment group after every 24 hours continuously for 2 two weeks.

#### 2.6.1. Gene Expression Analysis of Animal Infection and Treatment by qRT-PCR

Blood and feces samples were collected from each chicken and bacterial DNA was isolated from blood and feces of all the three groups of each bacteria using DNeasy Blood & Tissue Kit (QIAGEN, Waltham, Massachusetts, USA). DNA extraction from samples was done according to the manufacturer instruction of QI Aamp DNA stool minikit (QIAGEN). The isolated bacterial DNA was used as template and amplified by real time quantitative polymerase chain reaction (RT-PCR) using gen specific primers and the following conditions

15 *μ*L QuantiFast SYBR green master mix (Thermo Fisher Scientific), 1 *μ*L (10 *μ*M) primers each forward and reverse, 2 *μ*L template DNA, and DEPC treated water to make the final volume of 25 *μ*L. Cycles were set as initial denaturation 5 min at 95°C, 40 cycles of denaturation at 95°C for 30s, annealing at 60°C for 30s and followed by 30 extensions at 72°C. The product of PCR was visualized on agarose gel in the form of DNA bands.

### 2.7. Determination of Lysozyme Activity

The serum was obtained from chicken spleen following the method of Dai et al. 2014 [37]; lysozyme activity was measured by Lysozyme Activity Assay Kit (Fluorometric)-BioVision according to the manufacturer's protocol.

### 2.8. Statistical Analysis

Data are represented as means of experiment in triplicate ± SEM. SPSS 2l were used for statistical analyses. Analysis of data was made by one-way ANOVA followed by the Tukey-Kramer test as post hoc analysis. The level of significance was kept p < 0.05 for all statistical tests.

## 3. Results

**Figure 1:**
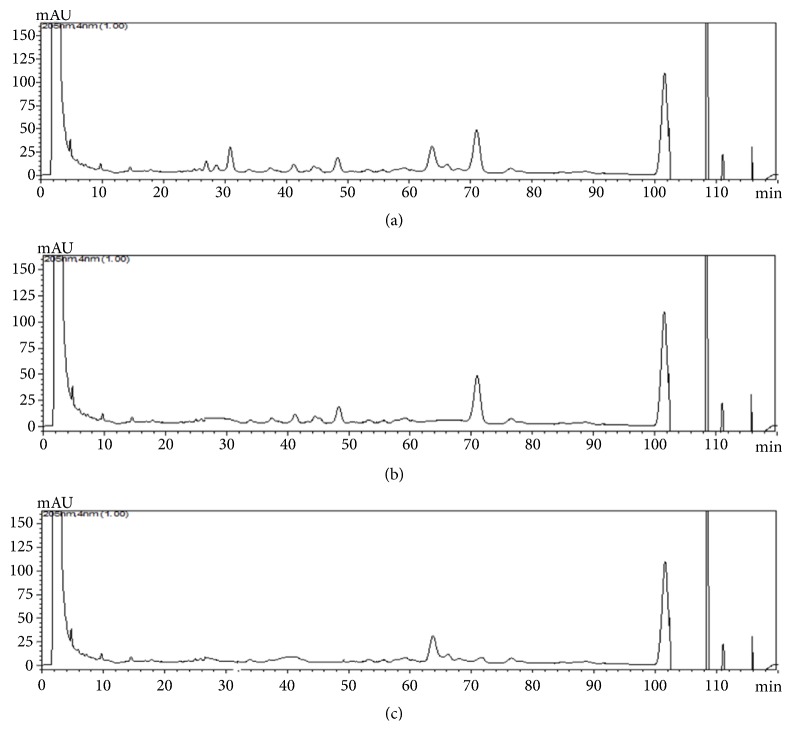
HPLC analysis of green tea seed isolated saponins fractions. Various saponins were detected in the saponins isolated fraction. (a) HPLC chromatogram of the saponin Fr1 of green tea seeds. (b) HPLC chromatogram of the saponin Fr2 of green tea seeds. (c) HPLC chromatogram of the saponin Fr3 of green tea seeds.

**Figure 2:**
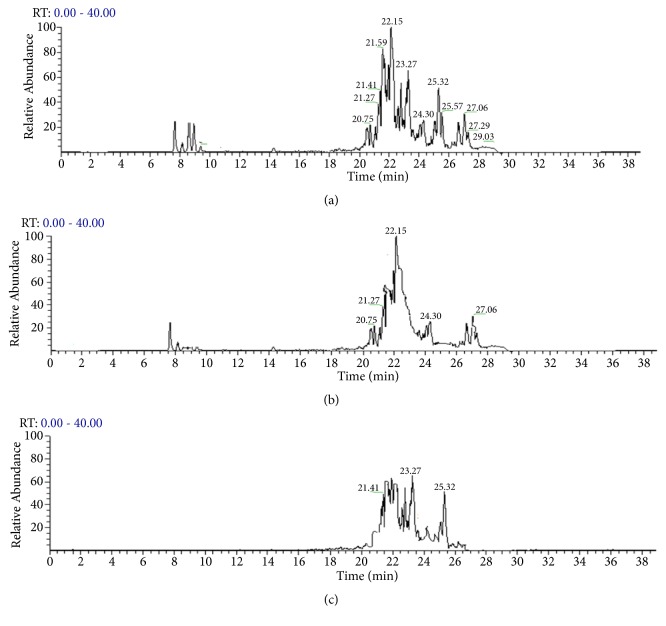
LC-MS analysis of green tea seed isolated saponins fractions. Various saponins were detected in the saponins isolated fraction. The major saponins identified are Theasaponin E1, Theasaponin C, Assamsaponin A (C57H88O25), Theasaponin E3 (C57H88O26), Theasaponin A1 (C57H90O26), Assamsaponin B (C61H92O28), and Theasaponin A3 (C61H94O28). (a) LC-MS spectra of the saponin Fr1 of green tea seeds. (b) LC-MS spectra of the saponin Fr2 of green tea seeds. (c) LC-MS spectra of the saponin Fr3 of green tea seeds.

The saponin fractions obtained were analyzed by LC-MS and NMR to identify and characterize the present saponins (Figures 1 and 2). The major saponins detected in Fr1 were Theasaponin E1(C_59_H_90_O_27_), Theasaponin C, Assamsaponin A (C_57_H_88_O_25_), Theasaponin E3 (C_57_H_88_O_26_), Theasaponin A1 (C_57_H_90_O_26_), Assamsaponin B (C_61_H_92_O_28_), and Theasaponin A3, (C_61_H_94_O_28_).

**Figure 3:**
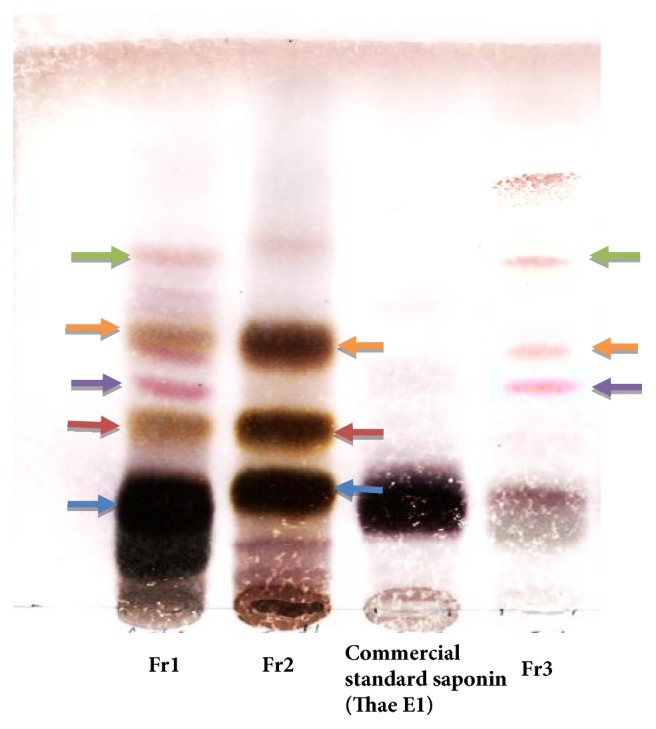
Thin-layer chromatography (TLC) of the isolated saponins fractions. Results were compared with the commercial grade Theasaponin E1. Same color arrows represent same type of saponins in different fraction.

Fr2 and Fr3 are obtained from Fr1 by preparative HPLC (Develosil ODS-HG-5, MeCN-0.05% aqueous TFA 55:45, 4 mL/min); Fr2 contained Theasaponin E1, Theasaponin A, and Theasaponin E3; and Fr3 contained Assamsaponin A, Theasaponin E3, and Assamsaponin B; the saponins were also analyzed by thin-layer chromatography (TLC). And the results were compared with commercial grade standard Theasaponin E1. Results show presence of Theasaponin E1 in the isolated fractions Fr1 and Fr2 (Figure 3)

### 3.1. In Vitro Antibacterial Assay

**Figure 4:**
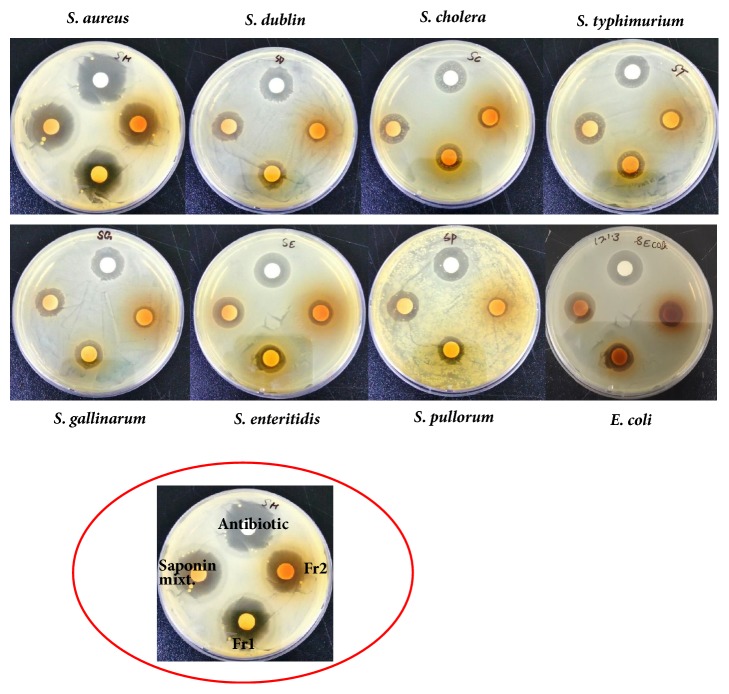
Growth inhibition of various bacteria strains by saponins fractions determined by disc-diffusion methods. Each plate contained 4 paper disks impregnated in 1 mg/ml solutions of each sample. Clear zone was measured for each samples against each strain. Ampicillin was used as standard. Experiments were performed in triplicate.

**Figure 5:**
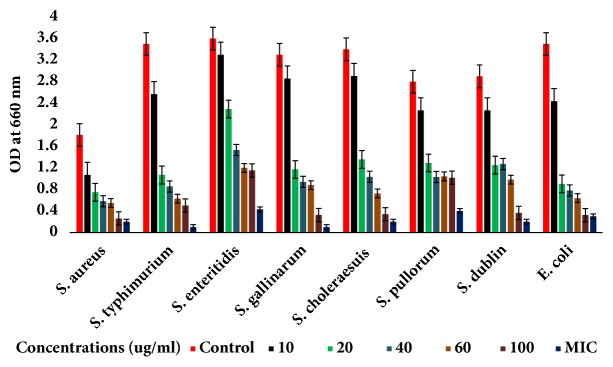
Antibacterial activities of Fr1 of green tea seed saponins against* Escherichia coli, Salmonella typhimurium, Salmonella enteritidis, Salmonella gallinarum, Salmonella choleraesuis, Salmonella pullorum, Salmonella dublin, and Staphylococcus aureus*. The antibacterial activities were determined by allowing the bacteria to grow in the presence of various concentrations of saponins using 96-well microtiter plate and then checking the viability of bacteria by measuring OD_660_ with spectrophotometer. Data are expressed as means of experiments in triplicate ± SEM. Data are statistically significant at P < 0.05.

**Figure 6:**
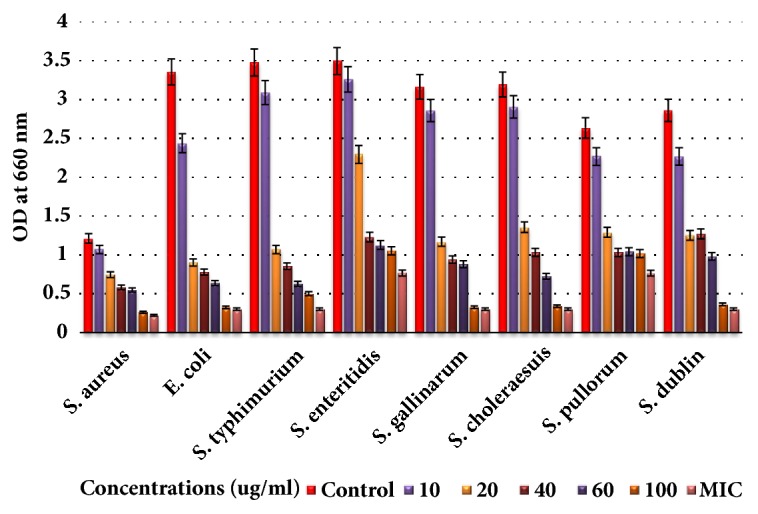
Antibacterial activities of Fr2 of green tea seed saponins against* Escherichia coli, Salmonella typhimurium, Salmonella enteritidis, Salmonella gallinarum, Salmonella choleraesuis, Salmonella pullorum, Salmonella dublin, and Staphylococcus aureus*. The antibacterial activities were determined by allowing the bacteria to grow in the presence of various concentrations of saponins using 96-well microtiter plate and then checking the viability of bacteria by measuring OD_660_ with spectrophotometer. Data are expressed as means of experiments in triplicate ± SEM. Data are statistically significant at P < 0.05.

**Figure 7:**
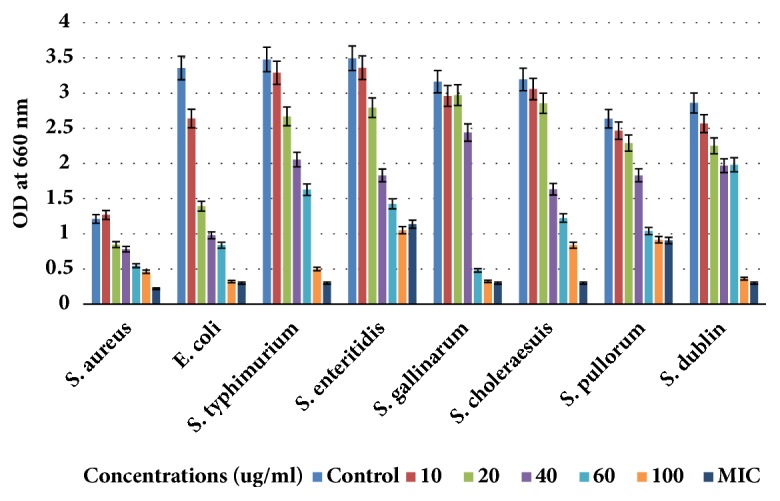
Antibacterial activities of saponin fraction Fr3 against* E. coli, Staphylococcus aureus, *and five serotypes of* Salmonella enterica*. The antibacterial activities were determined by allowing the bacteria to grow in the presence of various concentrations of saponin mixture using 96-well microtiter plate and then checking OD_660_ with spectrophotometer. Data are expressed as means of experiments in triplicate ± SEM. Data are statistically significant at P < 0.05.

The isolated saponins fractions from green tea seed were screened for their antibacterial potential. The preliminary antibacterial activities of saponin mixture and fractions against various bacteria were determined by disc-diffusion method. Agar plate was used for determination of the growth inhibition and clear zone formation against each bacterium. Each plate contained 4 paper disks impregnated in Fr1, Fr2, and Fr3 and the positive standard antibiotics ampicillins for comparison. The results indicated that the saponin fractions, i.e., inhibited visible growth of bacteria in different potential and range as shown in Figure 4. Fr1 is found to be more active against bacteria than Fr2 and Fr3. The clear zones on agar plates by the samples were measured as inhibition zones (IZ). The results indicated that the inhibition zones of the saponin fractions were diverse against different bacteria strains. However, the highest growth inhibition was made by Fr1 followed by Fr2 as compared to standard. Minimum inhibition concentrations were determined using 96-well plate against each bacterium (Table 1). The growth inhibitory effect and antibacterial activities of the saponins against* S. aureus, E. coli, *and different serotypes of the zoonosis causing* salmonella *were also measured by determining bacteria viability and lethality under saponins using 96-well microtiter plate. The results obtained show the effectiveness of the saponins against bacteria. The results show that the antibacterial activities of each sample were different against different bacteria; however highest antibacterial effects were observed in case of saponins Fr1 as compared to negative control (without saponins treatment). Highest growth inhibition was observed against* S. aureus *and lowest against* S. enteritidis; *also the antibacterial activities were found dose dependent, i.e., increasing with higher constrictions of the samples (Figures 5–7).

### 3.2. Antibacterial Mechanisms of Saponin/Bacterial Cell Lysis Potential of Saponins

**Figure 8:**
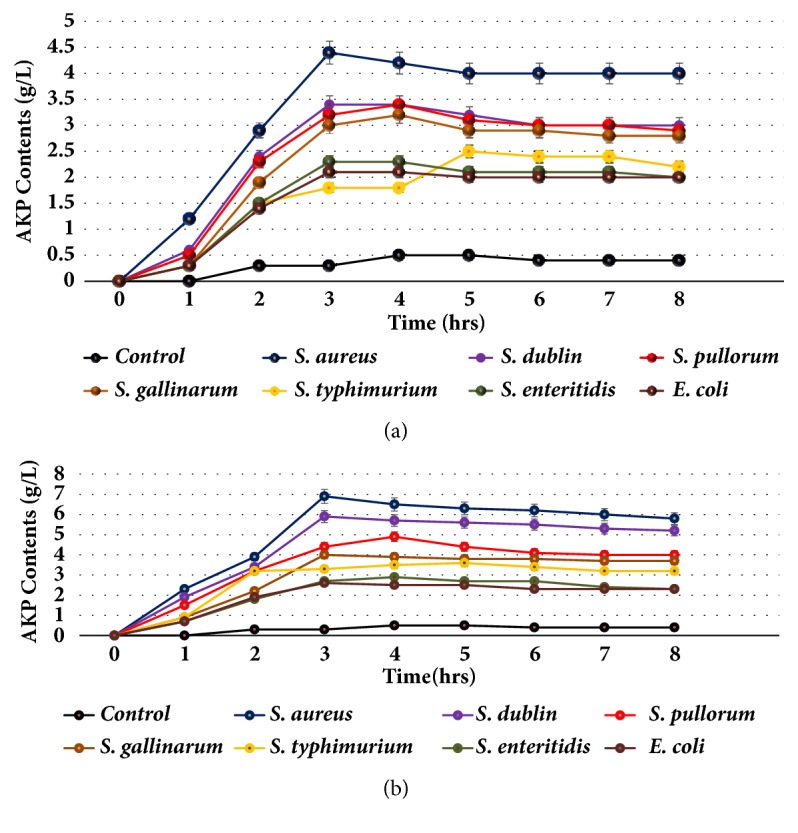
AKP (Alkaline phosphatase) contents after exposition of bacteria to saponins. Saponin caused damage to the bacterial cell wall and leakage of AKP to outside from the cell. (a) AKP contents of the group 100 ug/ml saponins treatment of bacteria. (b) AKP contents of the group treatment of bacteria with saponins at MIC.

Saponins caused lysis of bacterial wall as AKP contents were increased rapidly after the addition of saponins to the bacteria culture. The APK contents were found to be increased with increasing concentration of the saponins and it was higher in all cases as compared to control (Figure 8). AKP contents were highest at MIC after 3 hrs treatment in case of* S. aureus*. The results suggested lysis of bacterial cell by saponins and leakage of the AKP contents.

### 3.3. Effects on the Contents of Soluble Proteins of Bacteria

**Figure 9:**
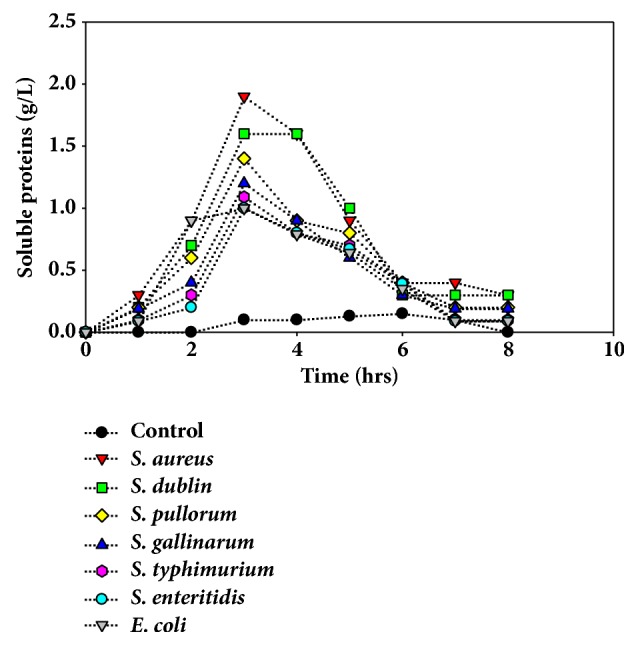
Effects of saponins on bacteria membrane determined by the leaked proteins contents at different time intervals after exposition of bacteria to saponins. Data are expressed as means of experiments in triplicate ± SEM.

The contents of soluble proteins were found to be increased with the increasing concentration of the saponins (Figure 9). The concentrations of soluble proteins were maximum for strain* S. aureus* and minimum for strain* E. coli* at 2 h. For all groups proteins contents were higher as compared to the control group. The results demonstrated that saponins may cause bacterial lysis or damage to the cell wall and member and lead to liberation of internal proteins contents to the external media. The higher amount of proteins contents represents higher lysis and damage of bacteria cells by saponins. Proteins contents were found to be decreased with time as used by bacteria.

### 3.4. In Vivo Antibacterial Assay

**Figure 10:**
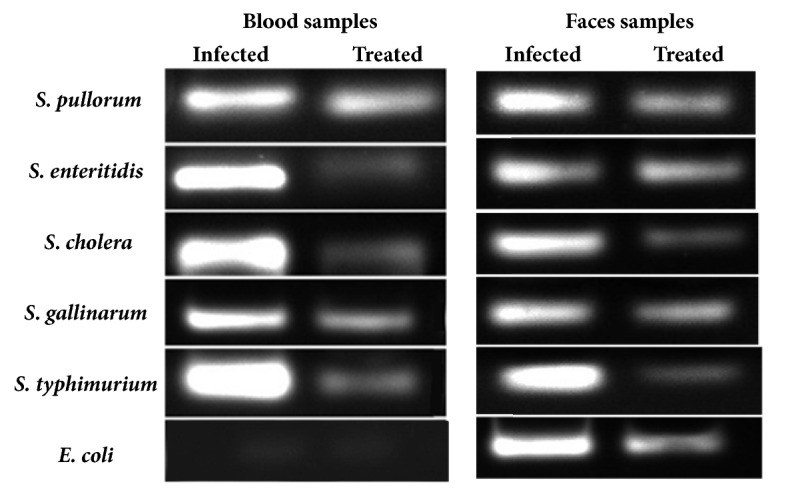
Detection of bacteria in the blood and feces samples of the infected group chickens and infected and treated group chickens by qRT-PCR analysis. Expression levels of the gens of different bacterial strains in the blood and feces samples. Results are confirmed by performing the experiments three times.

The blood and fecal samples taken from the chicken of all groups were analyzed for the presence of bacteria in the chickens. The DNA was extracted from the samples we amplified by real time quantitative PCR using gene specific primers for each salmonella (Table 2). The results demonstrated the presence of bacteria in the samples from infected groups with higher expressions levels of the genes. While the expressions levels of bacterial sequencing in samples of treatment groups were lower indicating the lower number of bacteria as treated with saponins (Figure 10), no bacteria were detected in the blood and feces samples of control group.

### 3.5. Lysozyme Activities

**Figure 11:**
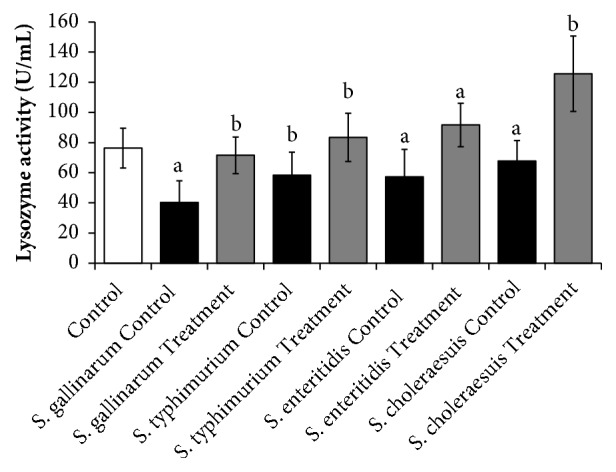
The immunity enhancing effect of the green tea seed extracted saponins mixture determined by lysozyme activity of the spleen isolated samples against the poultry infecting* salmonella* serotypes. Data are expressed as means of experiments in triplicate ± SEM. Data are statistically significant at P < 0.05. (a= P < 0.05, b= P < 0.01).

Effect of saponins on immunity of the animals was determined by lysozyme activity. Lysozyme or muramidase is the antimicrobial enzyme produced by animals as a part of innate immune system. The results show that the green tea seed extracted saponins are potent in increasing the lysozyme activities of animals in different range against different bacteria. Highest lysozyme activities were detected against* S. chloresus* while lowest were found against* S. gallinarum *as shown in Figure 11

## 4. Discussion

Antibiotics are substances that inhibit or kill the microorganisms. Antibiotics are quite important and very necessary in order to provide a feasible solution to control or inhibit the pathogenic bacteria. Bacteria resistivity and adverse side effects of synthetic antibiotics chemicals and search of novel antibiotics from natural sources are highly recommended. Natural products are comparatively safe, effective, and crucial materials for maltipurpose use and applications. Saponins are one of the diverse groups of plant sources compounds with valuable medial values and bioactivates. Saponins possess detergent-like properties and might increase the permeability of bacterial cell membranes; this activity might facilitate antibiotic influx through the bacterial cell wall membrane [38]. Saponins extracts from* Quillaja saponaria* are in use as foaming agents in beverages or emulsifiers in foods [39]. In present study we investigated the antibacterial activities of green tea (*Camellia sinensis*) derived saponins against Gram-positive and Gram-negative bacteria. The in vivo and* in vitro* antimicrobial activities of saponins from camellia seeds have not been described in detail, although we previously described antimicrobial activities of* C. sinensis* seeds derived crude saponins [21]. Our results confirmed that green tea isolated saponins possess strong antimicrobial activities. The isolated fractions Fr1 showed highest antibacterial activities as compared to Fr2 and Fr3. Antibacterial activity of saponins from some plant sources has been already reported [40, 41]. The green tea saponins were diverse in antibacterial effects against various bacterial strains. Different MIC values and IZ were calculated against different strains. MIC values were lower for Fr1as compared to Fr2 and Fr3. The inhibition zone (IZ) values were comparatively higher in case of Fr1 due to the presence of various types of major saponins. Similarly, the Fr1 showed comparatively higher activities in the bacteria lethality and viability assay. The antibacterial effects of the saponins were higher against* S. aureus* (Gram-positive bacteria) and comparatively lower in case of* S. entritidis* and* E. coli* (Gram-negative bacteria). Hence Fr1 showed comparatively higher antibacterial activities* in vitro* that is why this fraction was used further for investigating the lysis potential and* in vivo* antibacterial activities. Fr1 saponins were found effective in damaging cell wall of bacteria in dose-dependent manner determined by measuring the liberated soluble proteins and APK contents. Saponin damaged the bacteria wall with different potential for each bacteria strain. Lysozyme is an integral part of normal and nonspecific immunity. The molecules functions are protection against bacteria attack and considered to be a potent innate immunity molecule found in microorganisms, animals, and plants [42]. In the present study, the effects of green tea isolated saponins mixture on lysozyme activity in serum were evaluated compared with the normal control group, the activities of lysozyme in the saponins feed are significantly enhanced (p < 0.05). The results suggested that saponins could reinforce the immune function by raising the activity of lysozyme.

## 5. Conclusion

In conclusion, we demonstrated that saponins from green tea seed exhibit significant antibacterial activities* in vitro* and* in vivo* and increase the immune function by enhancing the activities of lysozyme. Green tea seed contained different types of saponins which exerts antibacterial activities against various strains of bacteria and specifically the zoonosis causing and infections spreading* salmonella*. The antibacterial activities are diverse depending on the types of saponin and the bacteria strains. Hence green tea saponins are best candidate for novel, effective, and natural antibiotic formulation.

## Figures and Tables

**Table 1:** Inhibition zones (IZ) and minimum inhibition concentration (MIC) values of saponins mixture, Fr1 and Fr2 against *S. aureus *(gram-positive), *E. coli *(gram-negative), and serovars of *salmonella* (gram-negative).

**Bacteria strains**	**Saponins mixt.**		**Fr1**		**Fr2**		**Standard**	
	**IZ**	**MIC**	**IZ**	**MIC**	**IZ**	**MIC**	**IZ**	**MIC**
*S. aureus*	12 ± 0.09^a^	0.15	11.3 ± 0.10^a^	0.20	10.3 ± 0.17^b^	0.25	15 ± 0.12^a^	0.10
*E. coli*	10.5 ± 0.22^a^	0.2	10 ± 1.33^b^	0.20	9 ± 0.13^c^	0.25	12.5 ± 0.17^b^	0.15
*S. typhimurium*	11.4 ± 0.31^c^	0.20	9.2 ± 0.20^c^	0.20	10.2 ± 0.20^c^	0.25	14.6 ± 0.13^c^	0.15
*S. enteritidis*	9.8 ± 0.42^b^	0.25	7.8 ± 0.067^a^	0.25	8.9 ± 0.21^a^	0.25	12.4 ± 0.08^b^	0.20
*S. gallinarum*	10.0 ± 0.45^a^	0.25	9.4 ± 0.32^c^	0.25	9.6 ± 0.20^a^	0.30	13 ± 0.14^a^	0.20
*S. choleraesuis*	11.3 ± 0.32^c^	0.30	10.3 ± 0.23^a^	0.30	11.4 ± 0.19^b^	0.35	13 ± 0.29^c^	0.20
*S. pullorum*	10.8 ± 0.31^b^	0.35	11.0 ± 0.12^b^	0.35	10.0 ± 0.23^c^	0.35	12 ± 0.20^a^	0.25
*S. dublin*	9 ± 0.41^b^	0.35	7.0 ± 0.26^c^	0.35	7.8 ± 0.21^b^	0.35	11 ± 0.13^c^	0.25

Inhibition zones (IZ)= mm.

Minimum inhibition concentration (MIC)= mg/ml.

a= *p*< 0.005, b= *p*<0.01, and c= *p*<0.05.

**Table 2:** Primers sequences of *E. coli* and various serotypes of *salmonella* used for in vivo antibacterial investigation of green tea seed extracted saponins.

**Genes**	**Strains**	**Primers**	
		**Forward**	**Reverse**
*uspA*	*E. coli*	CCGATACGCTGCCAATCAGT	ACGCAGACCGTAGGCCAGAT
*sefA*	*Salmonella enteritidis*	GCAGCGGTTACTATTGCAGC	CTGTGACAGGGACATTTAGCG
Serotype-d	*Salmonella typhimurium*	CCTTTCTCCATCGTCCTGAA	TGGTGTTATCTGCCTGACCA
rfbSP	*Salmonella pullorum*	GATCGAAAAAATAGTAGAATT	GCATCAAGTGATGAGATAATC
rfbSG	*Salmonella gallinarum*	GTATGGTTATTAGACGTTGTT	TATTCACGAATTGATATACTC
*fliC*	*Salmonella choleraesuis*	AAGGAAAAGATCATGGCACAA	GAACCCACCATCAATAACTTTG

## Data Availability

All of the data are in the manuscript and suporting matterials.

## Supplementary Materials

Supplementary Materials HPLC-Ms (Excel Files) and NMR data (PDF File) of the green tea seed saponins used in this study. 1: toxicity determination of saponins. Cells viability under various concentrations of green tea seed extracted saponins mixture using various cell lines and chickens. 2: NMR data of the green tea seed isolated saponins. 3: HPLC-MS full scan data of the green tea seed extracted saponins mixture compared to standard. 4: detection of various saponins in the green tea seed extracted saponins mixture by HPLC-MS analysis.
