# Supplementary material for: Green Tea Seed Isolated Saponins Exerts Antibacterial Effects against Various Strains of Gram Positive and Gram Negative Bacteria, a Comprehensive Study In Vitro and In Vivo
Source: Evid Based Complement Alternat Med. 2018 Nov 26;2018:3486106. doi: 10.1155/2018/3486106 (PMC6287149; doi:10.1155/2018/3486106)
Supplement: Supplementary Materials — HPLC-Ms (Excel Files) and NMR data (PDF File) of the green tea seed saponins used in this study. 1: toxicity determination of saponins. Cells viability under various concentrations of green tea seed extracted saponins mixture using various cell lines and chickens. 2: NMR data of the green tea seed isolated saponins. 3: HPLC-MS full scan data of the green tea seed extracted saponins mixture compared to standard. 4: detection of various saponins in the green tea seed extracted saponins mixture by HPLC-MS analysis. [file 3486106.f1.zip › 3486106.f1/NMR- data of isolated saponins.pdf]

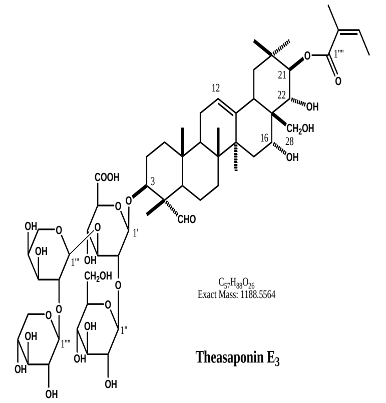


**Teasaponin E3**

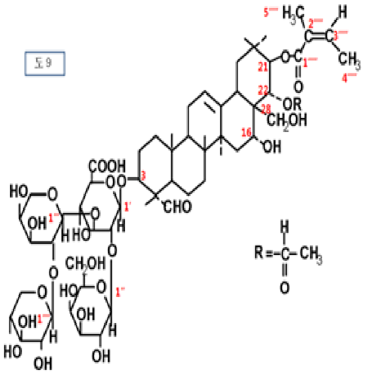


**Teasaponin E1(GTS E1)**

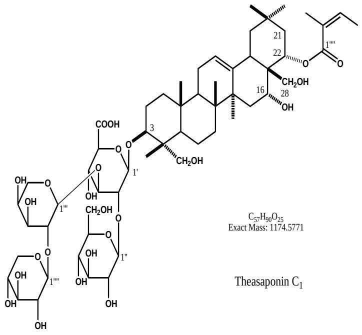


**Teasaponin C1**

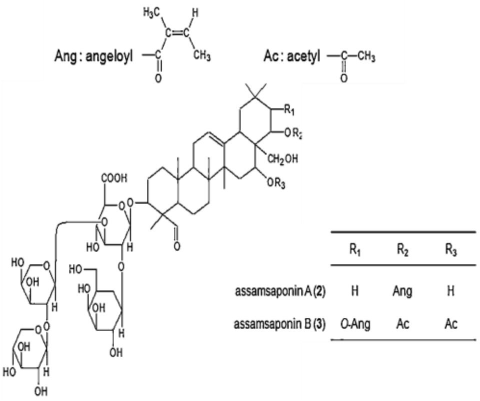


| | $R_1$ | $R_2$ | $R_3$ |
|---|---|---|---|
| assamsaponin A (2) | H | Ang | H |
| assamsaponin B (3) | O-Ang | Ac | Ac |

# assamsaponin A & B

**Theasaponin E3 NMR data**

| 표 4.$^{13}$C-NMR data of 544-3 in pyridine-$d_5$ | | | | | |
|---|---|---|---|---|---|
| Position | 544-3 | Theasaponin $E_3$[a] | Position | 544-3 | Theasaponin $E_3$[a] |
| 1 | 38.5 | 38.2 | 1′ | 104.5 | 104.2 |
| 2 | 25.6 | 25.3 | 2′ | 78.5 | 78.3 |
| 3 | 84.4 | 84.1 | 3′ | 84.9 | 84.5 |
| 4 | 55.5 | 55.2 | 4′ | 71.4 | 70.8 |
| 5 | 48.7 | 48.2 | 5′ | 77.5 | 77.3 |
| 6 | 20.7 | 20.4 | 6″ | 171.1 | 172.0 |
| 7 | 32.7 | 32.4 | 1″ | 103.6 | 103.3 |
| 8 | 40.6 | 40.3 | 2″ | 74.0 | 73.7 |
| 9 | 47.1 | 46.9 | 3″ | 75.7 | 75.4 |
| 10 | 36.4 | 36.2 | 4″ | 70.8 | 70.4 |
| 11 | 24.1 | 23.8 | 5″ | 76.9 | 76.5 |
| 12 | 123.4 | 123.1 | 6″ | 62.4 | 62.1 |
| 13 | 143.1 | 143.6 | 1‴ | 101.9 | 101.7 |
| 14 | 42.1 | 41.9 | 2‴ | 82.3 | 82.4 |
| 15 | 34.9 | 34.4 | 3‴ | 73.7 | 73.4 |
| 16 | 68.2 | 67.8 | 4‴ | 68.6 | 68.3 |
| 17 | 47.6 | 47.8 | 5‴ | 66.7 | 66.6 |
| 18 | 40.8 | 40.5 | 1′′′′ | 107.4 | 107.1 |
| 19 | 47.4 | 47.0 | 2′′′′ | 75.7 | 75.9 |
| 20 | 36.3 | 36.1 | 3′′′′ | 78.5 | 78.3 |
| 21 | 81.5 | 81.7 | 4′′′′ | 71.1 | 70.8 |
| 22 | 73.6 | 73.1 | 5′′′′ | 67.8 | 67.5 |
| 23 | 210.4 | 209.9 | 1′′′′′ | 168.9 | 168.7 |
| 24 | 11.4 | 11.1 | 2′′′′′ | 129.8 | 129.6 |
| 25 | 16.1 | 15.8 | 3′′′′′ | 136.3 | 136.0 |
| 26 | 17.3 | 16.9 | 4′′′′′ | 16.2 | 15.9 |
| 27 | 27.7 | 27.4 | 5′′′′′ | 21.4 | 21.1 |
| 28 | 66.3 | 66.0 | 1′′′′′′ | 64.3 | 64.5 |
| 29 | 30.1 | 29.9 | | | |
| 30 | 20.6 | 20.4 | | | |

## Theasaponin E1 NMR data

**표 5.$^{13}$C-NMR data of 544-4 in pyridine-$d_5$.**

| Position | 544-4 | reference | Position | 544-4 | reference |
|---|---|---|---|---|---|
| 1 | 38.5 | 38.3 | 1′ | 104.4 | 104.1 |
| 2 | 25.5 | 25.2 | 2′ | 78.6 | 78.4 |
| 3 | 84.8 | 84.5 | 3′ | 84.1 | 84.2 |
| 4 | 55.4 | 55.2 | 4′ | 71.2 | 70.8 |
| 5 | 48.7 | 48.4 | 5′ | 76.9 | 77.3 |
| 6 | 20.7 | 20.4 | 6″ | 171.3 | 171.8 |
| 7 | 32.7 | 32.5 | 1″ | 103.6 | 103.2 |
| 8 | 40.6 | 40.4 | 2″ | 74.0 | 73.7 |
| 9 | 47.1 | 46.8 | 3″ | 75.7 | 75.3 |
| 10 | 36.4 | 36.1 | 4″ | 70.8 | 70.5 |
| 11 | 24.1 | 23.8 | 5″ | 76.8 | 76.5 |
| 12 | 123.4 | 123.1 | 6″ | 62.4 | 62.1 |
| 13 | 143.3 | 142.9 | 1‴ | 102.0 | 101.7 |
| 14 | 42.0 | 41.8 | 2‴ | 82.3 | 82.3 |
| 15 | 34.9 | 34.6 | 3‴ | 73.8 | 73.4 |
| 16 | 68.2 | 68.1 | 4‴ | 68.7 | 68.4 |
| 17 | 48.3 | 48.0 | 5‴ | 66.5 | 66.1 |
| 18 | 40.4 | 40.2 | 1⁗ | 107.4 | 107.0 |
| 19 | 47.5 | 47.2 | 2⁗ | 76.3 | 75.9 |
| 20 | 36.6 | 36.3 | 3⁗ | 78.5 | 78.2 |
| 21 | 79.2 | 78.9 | 4⁗ | 70.8 | 70.8 |
| 22 | 74.6 | 74.5 | 5⁗ | 68.0 | 67.5 |
| 23 | 210.3 | 209.8 | 1′′′′′ | 168.2 | 167.9 |
| 24 | 11.4 | 11.0 | 2′′′′′ | 129.3 | 129.0 |
| 25 | 16.1 | 15.8 | 3′′′′′ | 137.5 | 137.0 |
| 26 | 17.1 | 16.9 | 4′′′′′ | 16.3 | 15.9 |
| 27 | 27.7 | 27.4 | 5′′′′′ | 21.4 | 21.0 |
| 28 | 64.1 | 64.0 | 1′′′′′′ | 170.4 | 171.1 |
| 29 | 29.8 | 29.5 | 2′′′′′′ | 21.2 | 20.9 |
| 30 | 20.6 | 20.3 | | | |

## Theasaponin C1 NMR data

**표 6. $^{13}$C-NMR data of 551G3-1 in pyridine-$d_5$.**

| Position | 551G3-1 | Theasaponin $C_1$[a] | Position | 551G3-1 | Theasaponin $C_1$[a] |
|---|---|---|---|---|---|
| 1 | 39.0 | 38.7 | 1′ | 104.4 | 104.1 |
| 2 | 25.8 | 25.5 | 2′ | 78.8) | 78.5 |
| 3 | 83.3 | 83.1 | 3′ | 84.2 | 84.6 |
| 4 | 43.8 | 43.5 | 4′ | 70.5 | 71.0 |
| 5 | 48.4 | 48.2 | 5′ | 77.1 | 77.4 |
| 6 | 18.4 | 18.2 | 6″ | 172.3 | 172.0 |
| 7 | 33.1 | 32.8 | 1″ | 103.4 | 103.2 |
| 8 | 40.4 | 40.1 | 2″ | 74.0 | 73.8 |
| 9 | 47.3 | 47.0 | 3″ | 75.5 | 75.3 |
| 10 | 37.0 | 36.8 | 4″ | 70.4 | 70.1 |
| 11 | 24.1 | 23.9 | 5″ | 76.8 | 76.5 |
| 12 | 123.4 | 123.1 | 6″ | 62.2 | 61.9 |
| 13 | 144.0 | 143.7 | 1‴ | 101.9 | 101.7 |
| 14 | 41.9 | 41.6 | 2‴ | 82.6 | 82.3 |
| 15 | 35.4 | 35.2 | 3‴ | 73.7 | 73.4 |
| 16 | 70.4 | 70.1 | 4‴ | 68.6 | 68.3 |
| 17 | 45.1 | 44.8 | 5‴ | 66.9 | 66.6 |
| 18 | 41.2 | 40.9 | 1′′′′ | 107.3 | 107.1 |
| 19 | 47.7 | 47.4 | 2′′′′ | 76.2 | 75.9 |
| 20 | 32.3 | 32.1 | 3′′′′ | 78.5 | 78.3 |
| 21 | 42.0 | 41.7 | 4′′′′ | 71.1 | 70.8 |
| 22 | 73.3 | 73.0 | 5′′′′ | 67.8 | 67.5 |
| 23 | 65.1 | 64.8 | 1′′′′′ | 168.3 | 168.0 |
| 24 | 13.9 | 13.6 | 2′′′′′ | 129.8 | 129.5 |
| 25 | 16.5 | 16.2 | 3′′′′′ | 136.9 | 136.6 |
| 26 | 17.2 | 16.9 | 4′′′′′ | 16.2 | 15.9 |
| 27 | 27.9 | 27.6 | 5′′′′′ | 21.3 | 21.0 |
| 28 | 63.9 | 63.6 | | | |
| 29 | 33.7 | 33.5 | | | |
| 30 | 25.5 | 25.2 | | | |

NMR data of Assamsaponin A and Assamsaponin B

| Assamsaponin A | | Assamsaponin B | |
|---|---|---|---|
| $\delta_C$ (ppm) | $\delta_H$ (ppm, *J*) | $\delta_C$ (ppm) | $\delta_H$ (ppm, *J*) |
| 37.9 | 1.53 (m) | 37.5 | 1.53(m) |
| 23.6 | 1.51 (m) | 24.6 | 1.48 (m) |
| 81.3 | 3.62 (m) | 73.4 | 3.59 (m) |
| 21.0 | | 22.3 | |
| 48.2 | 1.25 (m) | 41.4 | 1.22 (m) |
| 19.6 | 1.37 (m) | 18.6 | 1.31 (m) |
| 31.4 | 1.45 (m) | 30.5 | 1.45 (m) |
| 39.3 | | 35.2 | |
| 45.7 | 1.57(m) | 41.7 | 1.59 (m) |
| 35.1 | | 34.6 | |
| 22.7 | 1.62 (m) | 23.8 | 1.61 (m) |
| 122.4 | 5.11 (br s) | 120.4 | 5.18 (m) |
| 140.8 | | 140.2 | |
| 40.3 | | 40.2 | |
| 31.3 | 1.49 (m) | 31.6 | 1.33 (m) |
| 15.4 | 0.82 (s) | 15.0 | 0.84 (s) |
| 14.3 | 0.81 (s) | 15.0 | 0.83 (s) |
| 25.9 | 1.32 (s) | 24.3 | 1.37 (s) |
| 62.8 | 3.6 (m), | 62.3 | 3.1 (m) |
| 33.4 | 1.85 (s) | 31.4 | 1.84 (s) |
| 24.3 | 2.96 (s) | 23.9 | 2.0 (s) |
| | | | |
| 101.3 | 4.1 (br s) | 101.3 | 4.16 (br s) |
| 73.7 | 3.41 (m) | 73.6 | 3.46 (m) |
| 59.2 | 2.51 (m) | 59.3 | 3.58 (m) |
| 79.4 | 2.62 (m) | 78.2 | 3.60 (m) |

| | | | |
|---|---|---|---|
| 65.8 | 3.61 (m) | 62.2 | 3.65 (m) |
| 171.1 | | 171.2 | |
| | | | |
| 101.7 | 4.31 (m) | 101.5 | 4.30 (m) |
| 71.4 | 3.11 (m) | 73.5 | 3.09 (m) |
| 73.5 | 3.26 (m) | 69.7 | 3.29 (m) |
| 68.1 | 3.45 (m) | 68.1 | 3.42 (m) |
| 74.6 | 3.31 (m) | 72.3 | 3.37 (m) |
| 59.8 | 3.56 (m) | 60.2 | 3.47 (m) |
| | | | |
| | | | |
| 69.3 | 3.29 (m) | 75.1 | 3.32 (m) |
| 74.6 | 3.37 (m) | 73.6 | 3.37 (m) |
| 69.5 | 3.47 (m) | 69.3 | 3.47 (m) |
| 81.6 | 3.58 (m) | 81.7 | 3.52 (m) |
| | | | |
| 104.6 | 4.29 (br s) | 104.1 | 4.32 (d, 7.27) |
| 69.3 | 3.29 (m) | 69.8 | 3.78 (m) |
| 76.4 | 3.10 (m) | 76.7 | 3.67 (m) |
| 73.8 | 3.00 (m) | 73.2 | 3.45 (m) |
| 65.7 | 3.06 (m) | 65.7 | 3.19 (m) |
| | | | |
| 165.4 | | 165.3 | |
| 126.3 | | 126.9 | |
| 112.4 | 5.01 (dq) | 112.5 | 6.08 (dq) |
| 12.3 | 1.89 (m) | 12.7 | 1.89 (m) |
| 25.3 | 1.79(s) | 25.3 | 1.78 (s) |
| | | | |
