# Supplementary material for: Green Tea Seed Isolated Saponins Exerts Antibacterial Effects against Various Strains of Gram Positive and Gram Negative Bacteria, a Comprehensive Study In Vitro and In Vivo
Source: Evid Based Complement Alternat Med. 2018 Nov 26;2018:3486106. doi: 10.1155/2018/3486106 (PMC6287149; doi:10.1155/2018/3486106)
Supplement: Supplementary Materials — HPLC-Ms (Excel Files) and NMR data (PDF File) of the green tea seed saponins used in this study. 1: toxicity determination of saponins. Cells viability under various concentrations of green tea seed extracted saponins mixture using various cell lines and chickens. 2: NMR data of the green tea seed isolated saponins. 3: HPLC-MS full scan data of the green tea seed extracted saponins mixture compared to standard. 4: detection of various saponins in the green tea seed extracted saponins mixture by HPLC-MS analysis. [file 3486106.f1.zip › 3486106.f1/Toxicity determination of saponins.pdf]

Cell viability assays

Toxic and safe doses of saponins were determined by MTT assay using human umbilical vein endothelial cell (HUVECs), 3T3-L1 Adipocytes and chickens directly.

1) HUVECs viability under various concentrations of saponins Fr1(mixture). Determined by MTT assay. Data are mean values (n=3) ±SEM. Data are statistically significant at P<0.05

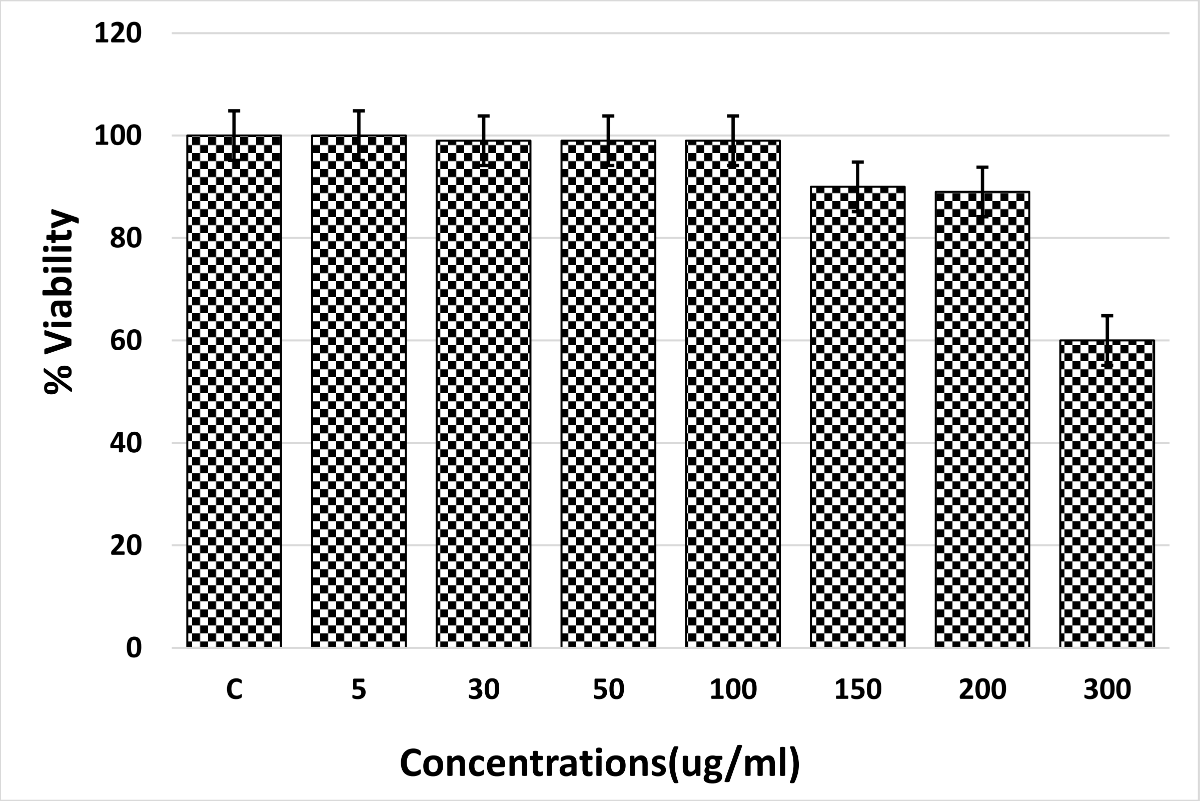

2) 3T3-L1 adipocytes viability under various concentrations of saponins Fr1(mixture). Determined by MTT assay. Data are mean values (n=3) ±SEM. Data are statistically significant at P<0.05

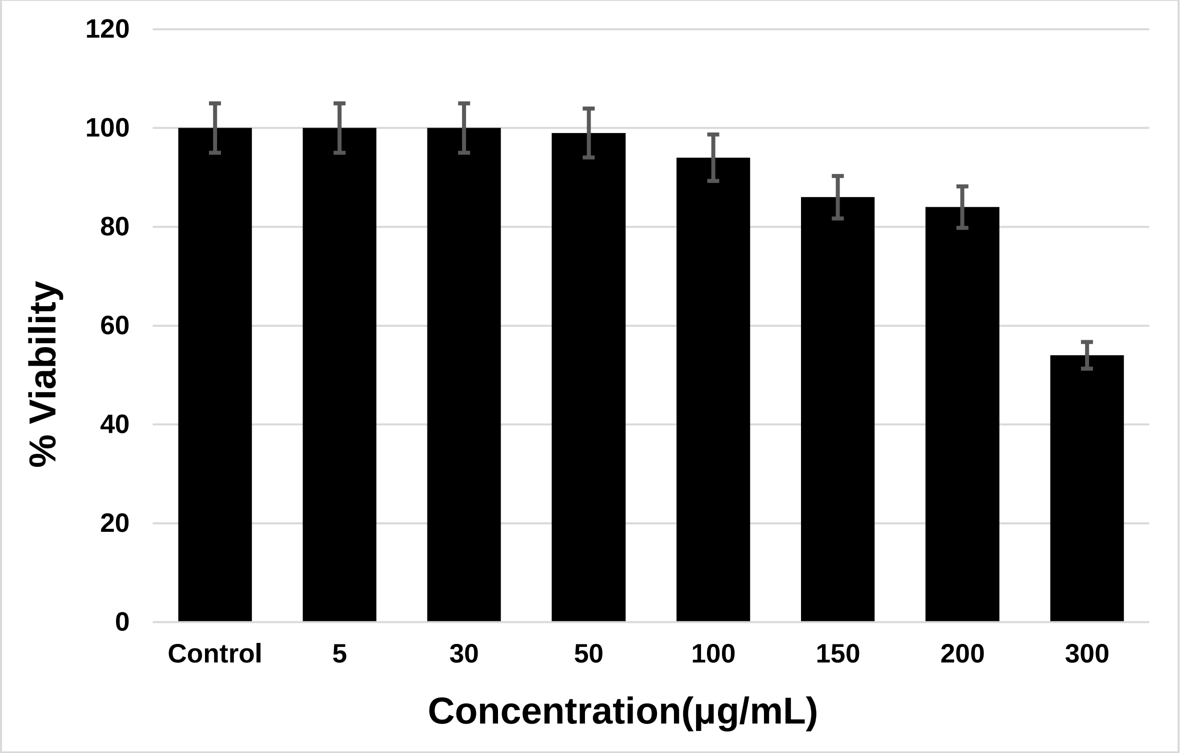

3. ***In vivo* toxicity determination of saponins mixture (Fr1) using chickens.**

The toxic and safe concentration level of saponins mixture was determined by 2 stages experiments as Level 1st and level 2nd experiments using same chickens in both levels. Saponins (aqueous solution) at different concentrations were orally administered to five different chicken used in duplicate in 5ml distilled water. The chickens were monitored for 72 hours for death (or diseased) or living status.

Level 1st experiment

Saponins Concentration(ug/5ml)	Chicken status	
	Chicken 1	Chicken 2
100	Healthy& Alive	Healthy& Alive
200	Healthy& Alive	Healthy& Alive
400	Healthy& Alive	Healthy& Alive
800	Healthy& Alive	Healthy& Alive
1000	Healthy& Alive	Healthy& Alive

2nd Level experiment

Saponins Concentration(mg/5ml)	Chicken status	
	Chicken 1	Chicken 2
2.0	Healthy& Alive	Healthy& Alive
4.0	Healthy& Alive	Healthy& Alive
6.0	Healthy& Alive	Healthy& Alive
8.0.	Healthy& Alive	Healthy& Alive
10.0	Healthy& Alive	died
